# Host Tree and Geography Induce Metabolic Shifts in the Epiphytic Liverwort *Radula complanata*

**DOI:** 10.3390/plants12030571

**Published:** 2023-01-27

**Authors:** Kaitlyn L. Blatt-Janmaat, Steffen Neumann, Jörg Ziegler, Kristian Peters

**Affiliations:** 1 Department of Chemistry, University of New Brunswick, Fredericton, NB E3B 5A3, Canada; 2 Bioinformatics and Scientific Data, Leibniz Institute of Plant Biochemistry, 06120 Halle (Saale), Germany; 3 German Centre for Integrative Biodiversity Research (iDiv) Halle-Jena-Leipzig, 04103 Leipzig, Germany; 4 Molecular Signal Processing, Leibniz Institute of Plant Biochemistry, 06120 Halle (Saale), Germany; 5 Institute of Biology/Geobotany and Botanical Garden, Martin Luther University Halle-Wittenberg, 06108 Halle (Saale), Germany

**Keywords:** ecological metabolomics, natural product chemistry, chemical ecology, bryophyte, liverwort, epiphyte

## Abstract

Bryophytes are prolific producers of unique, specialized metabolites that are not found in other plants. As many of these unique natural products are potentially interesting, for example, pharmacological use, variations in the production regarding ecological or environmental conditions have not often been investigated. Here, we investigate metabolic shifts in the epiphytic *Radula complanata* L. (Dumort) with regard to different environmental conditions and the type of phorophyte (host tree). Plant material was harvested from three different locations in Sweden, Germany, and Canada and subjected to untargeted liquid chromatography high-resolution mass-spectrometry (UPLC/ESI-QTOF-MS) and data-dependent acquisition (DDA-MS). Using multivariate statistics, variable selection methods, in silico compound identification, and compound classification, a large amount of variation (39%) in the metabolite profiles was attributed to the type of host tree and 25% to differences in environmental conditions. We identified 55 compounds to vary significantly depending on the host tree (36 on the family level) and 23 compounds to characterize *R. complanata* in different environments. Taken together, we found metabolic shifts mainly in primary metabolites that were associated with the drought response to different humidity levels. The metabolic shifts were highly specific to the host tree, including mostly specialized metabolites suggesting high levels of ecological interaction. As *R. complanata* is a widely distributed generalist species, we found it to flexibly adapt its metabolome according to different conditions. We found metabolic composition to also mirror the constitution of the habitat, which makes it interesting for conservation measures.

## 1. Introduction

Liverworts have been identified as prolific producers of unique, specialized metabolites that are not found in other plants, specifically metabolites that have pharmacological potential [1,2,3]. The majority of the specialized metabolite diversity in liverworts can be attributed to oil bodies, unique organelles which are not found in other bryophytes [4,5]. These structures are involved in the synthesis and storage of specialized metabolites that could be cytotoxic to the plant in large quantities [6,7,8]. The oil bodies, and by extension, the compounds within them, play a crucial role in defending the plant against herbivory. However, no specific antiherbivore metabolites from liverworts have been identified [9,10]. Many of the major compound classes found in the oil bodies have been found to respond greatly to environmental stimuli such as changes in micro-climatic conditions, humidity, the surrounding tree community, or pollution [6,11]. While pollution has been intensely investigated and has been found to have detrimental effects on the metabolism of epiphytic bryophytes [12], little work has been done to identify specific metabolite fluctuations with regard to other environmental changes. Fluctuations in these compounds may also be due to the substrate type and specific microclimate experienced by these plants, as flavonoid content is higher in epiphytic bryophytes than in terrestrial or aquatic species [13]. In addition to climatic factors which may be contributing to metabolite fluctuations, interactions with the host tree (phorophyte) may also be playing a role. Finally, for conservation, the presence or absence of certain epiphytic bryophytes (including liverworts) can predict the status of the habitat, which often warrants subsequent conservation measures. This is due to the remarkable capability of bryophytes to respond suddenly to environmental changes, and hence, many species act as distinctive bioindicators [14,15].

It is well documented that the phorophyte itself plays a role in shaping the epiphytic community that exists upon it, in addition to the microclimate surrounding it [16]. In general, the largest factor influencing the composition of epiphytic communities appears to be water availability, which can be impacted by both bark characteristics and microclimate [17]. In Western Scotland, the epiphytic communities of *Quercus* and *Fraxinus* were largely shaped by the height, angle, and texture of the tree bark [18]. The authors proposed that these factors emerged as the dominant ones due to their impact on the moisture level of the microclimate. In the Senchal Wildlife Sanctuary, a similar result was obtained when it was observed that older trees with rougher bark, wider bases, and better shading were preferred by epiphytic liverworts [19]. Bark pH and nutrient content also play a role in shaping epiphytic communities, specifically with regard to the abundance of lichens [20]. The fact that the chemistry of the host tree does not appear to drastically impact the community structure of these epiphytes is surprising, given how sensitive epiphytic bryophytes and lichens are to chemical disturbances, such as pollution [12,21,22].

While it is well established that epiphytic bryophyte communities are shaped by the microclimate and physical characteristics of the host tree they inhabit, there has been little research regarding the chemical response of the bryophytes to these different conditions. To determine if environmental conditions alter the metabolite profile of epiphytic liverworts, *Radula complanata* was selected as the target species. *R. complanata* is a leafy liverwort that is widely distributed in South America, North America, Europe, Asia, and northern Africa, making it an ideal candidate for studying environmental differences [23]. It prefers environments with high humidity and can utilize bark, rock, or dirt as a growth substrate. *R. complanata* also serves as a host for several different microorganisms, including *Bryocentria metzgeriae* (Ade & Hhn.) Döbbeler (a parasitic fungus), *Belonioscyphella hypnorum* (Syd. & P. Syd.) Hohn (a fungus that colonizes the plant in calcareous areas), various mite species, and different species of protozoa [23]. In this work, epiphytic *R. complanata* samples were collected from Canada, Germany, and Sweden during the summer/early fall of 2021, and the metabolite profiles were investigated to identify any metabolic shifts that are caused by the different environmental conditions.

## 2. Results

**Figure 1:**
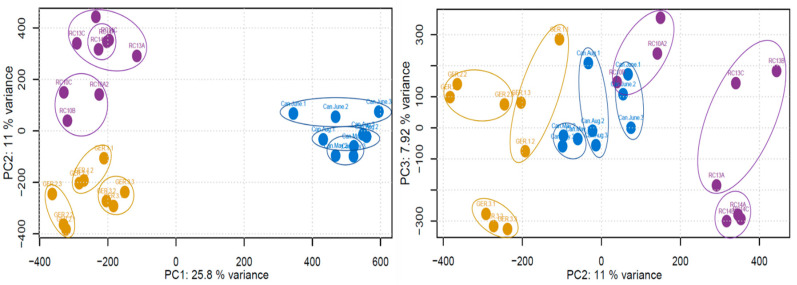
PCAs constructed using the raw MS1 data. Points are colored based on the sampled country (Purple—Sweden, Yellow—Germany, Blue—Canada), and circles have been added for clarity.

After the raw data was processed, a principal component analysis (PCA) was conducted on the MS1 data to assess the clustering of the samples (Figure 1). The country of origin appeared to be responsible for the variation observed across PC1 (25.8% variance), specifically based on the longitudinal location of the country. PC2 (11% variance) and PC3 (7.92% variance) were less clear, with the separation of the individual samples occurring across both principal components.

**Figure 2:**
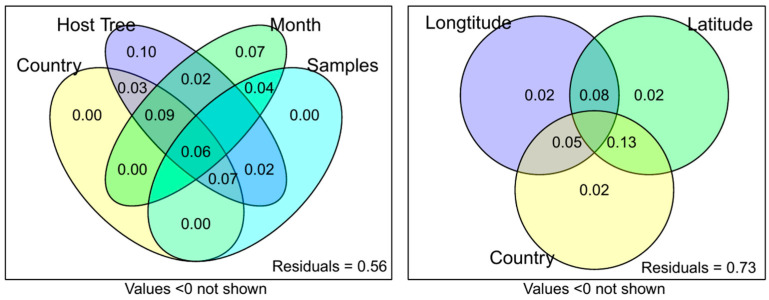
Variation partitioning of MS1 data to identify which factors may be contributing to the variation in the data.

To further elucidate the factors responsible for the clustering of samples observed in Figure 1, variation partitioning was conducted (Figure 2). The three main factors identified by the experimental design were country of origin, which could be further subdivided by longitude and latitude, the sampling month, and the phorophyte species (in the following referred to as host tree). All variation within the samples could be explained by these three factors, with 56% of the variation remaining unexplained. Interestingly, no unique variation was caused by the country of origin, while the host tree species accounted for 10% variation, and the sampling month accounted for 7% variation. A breakdown of the country factor showed that latitude shared more variation with the country (13%) than longitude did (5%).

**Figure 3:**
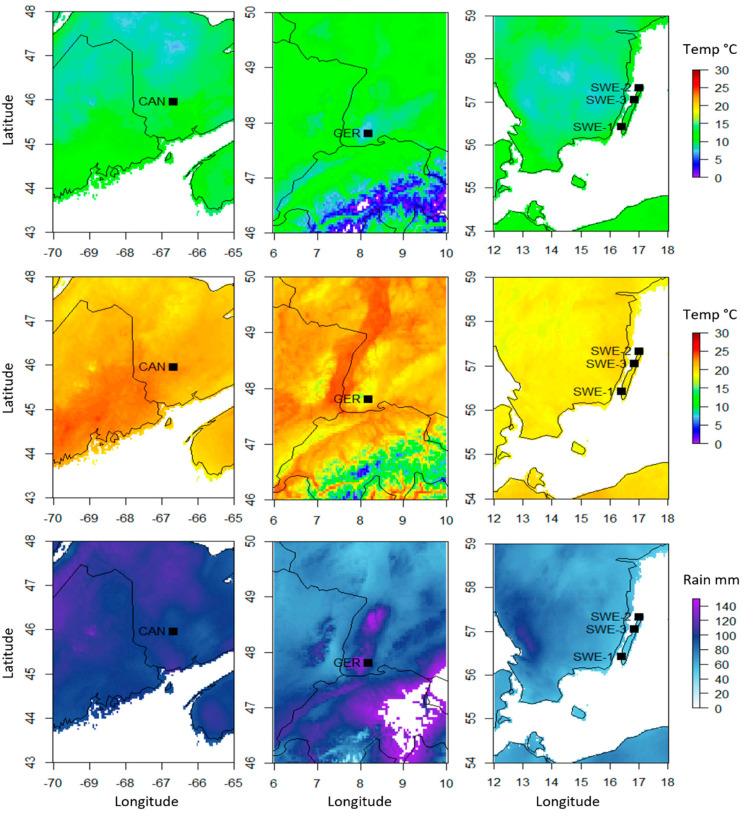
Average summer (May–September) temperature and precipitation data from the three sampled sites. Average minimum temperatures (**top**), maximum temperatures (**middle**), and monthly precipitation (**bottom**) data were compiled from 2010–2018 data available on WordClim. Individual samples from Canada and Germany samples were too close geographically to be distinguished, so one sample point from each country was used for figure clarity.

Weather maps of all three sampling sites were constructed to identify climatic factors that may contribute to the variation caused by the country factor (Figure 3, Table 1). Here, monthly weather data from WorldClim version 2.1 [24] was utilized. The sites in Germany had the lowest summer temperatures (7.69 °C to 18.45 °C), while the sites in Canada had the highest (10.35 °C to 23.09 °C and 10.66 °C to 23.49 °C). The sites in Sweden had much less rainfall (45.93 mm to 49.68 mm) than the sites in either Germany (115.97 mm) or Canada (97.40 mm and 97.76 mm). Older climate data (1970–2000) was consulted to compare the average solar radiation, wind speed, and water vapor pressure. Canada experienced the highest average solar radiation (17,869.2 kJ m-2 day-1) and the highest water vapor pressure (1.31 kPa and 1.302 kPa), while Sweden had the highest wind speeds (N/A, 4.52 m s^−1^, and 4.42 m s^−1^).

**Figure 4:**
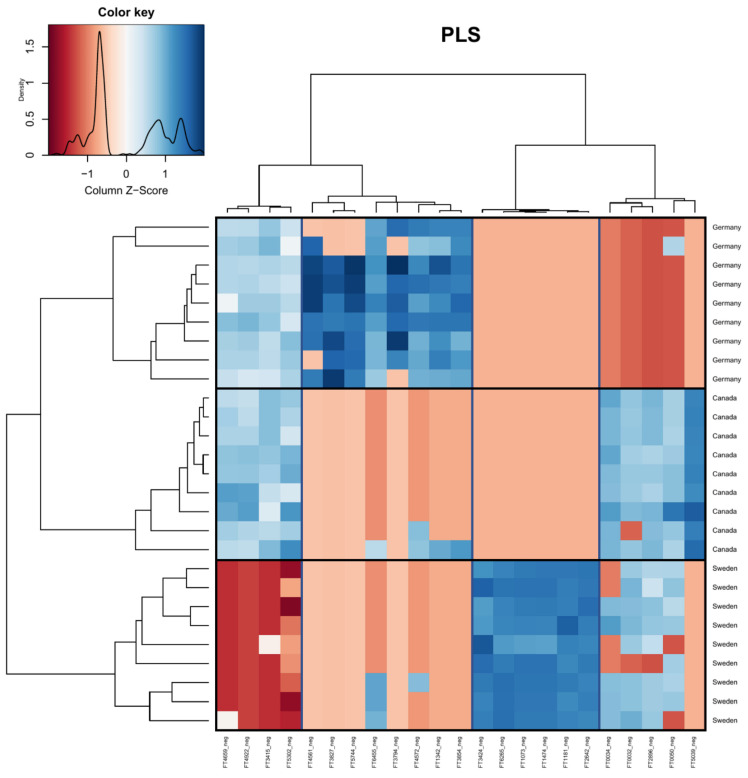
Heatmap visualizing the PLS of the compound table at the level of sample location (country). Boxes have been added for clarity (vertical = feature grouping, horizontal = sample grouping). Red indicates that peak intensities were low, while blue indicates that peak intensities were high. Identification of metabolite features is available in the Supplement (Table S1).

**Figure 5:**
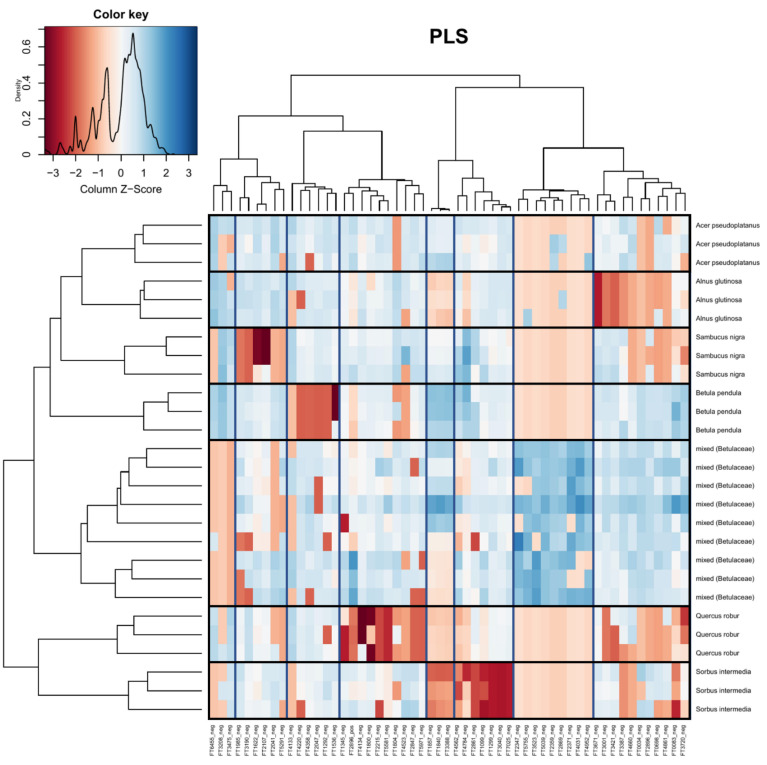
Heatmap visualizing the PLS of the compound table at the level of sample host tree (species). Boxes have been added for clarity (vertical = feature grouping, horizontal = sample grouping). Red indicates that peak intensities were low, while blue indicates that peak intensities were high. Identification of metabolite features is available in the Supplement (Table S2).

**Figure 6:**
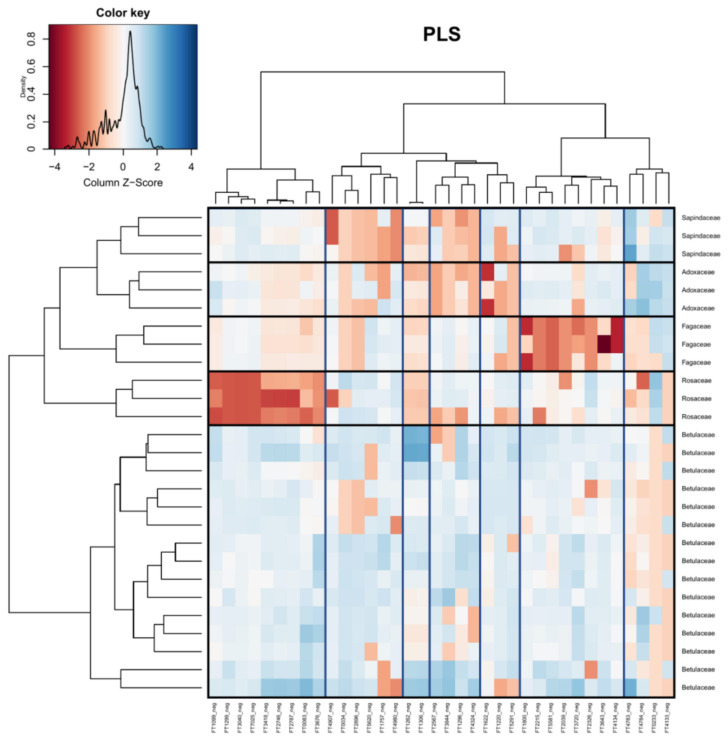
Heatmap visualizing the PLS of the compound table at the level of sample host tree (family). Boxes have been added for clarity (vertical = feature grouping, horizontal = sample grouping). Red indicates that peak intensities were low, while blue indicates that peak intensities were high. Identification of metabolite features is available in the Supplement (Table S3).

Partial least squares regressions (PLS) were conducted to identify the specific features responsible for the variation observed in the MS1 data. Figure 4 shows the PLS conducted at the level of “country”, Figure 5 shows the PLS conducted at the level of “host tree species”, and Figure 6 shows the PLS conducted at the level of “host tree family”. A PLS of the sampling month was conducted as well; however, no clear clustering of samples was observed based on the sampling month alone. In Figure 4, 20 out of the 23 selected features were successfully classified. Fifteen features were classified as primary metabolites, with the major classes being amino acids and peptides. The remaining five features belonged to specialized metabolite classes, including flavonoids, alcohols, and benzene derivatives. Interestingly, Germany and Canada clustered more closely than Germany and Sweden, which is inconsistent with the PCA but consistent with the variation portioning.

In Figure 5 (host tree species), 53 out of 55 features were successfully classified. Twenty-five features were classified as primary metabolites, with the major classes being amino acids and peptides. The remaining 28 features were classified as specialized metabolites, with the major classes being flavonoids and phenolic glycosides. Three compounds were tentatively identified: FT3190 was identified as Lysophosphatidylethanolamine(18:2/0:0), FT4562 was identified as Vicenin-2, and FT2347 was identified as Imbricaric acid.

In Figure 6 (host tree family), 33 out of 36 features were successfully classified. Eighteen features were classified as primary metabolites, with the major classes being amino acids and peptides. The remaining 15 features were specialized metabolite classes, including stilbenes, phenolic glycosides, flavonoids, and terpene glycosides. Two compounds were tentatively identified: FT3418 was identified as 2-[3,5-Dimethoxy-4-[3,4,5-trihydroxy-6-(hydroxymethyl)oxan-2-yl]oxyphenoxy]-6-(hydroxymethyl)oxane-3,4,5-triol and FT2567 was identified as 6-[3,5-Dihydroxy-2-(3-phenylpropanoyl)phenoxy]-3,4,5-trihydroxyoxane-2-carboylic acid.

**Figure 7:**
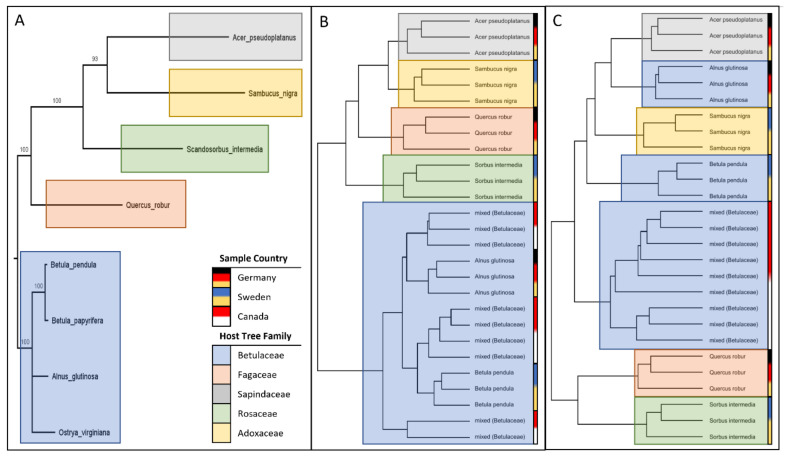
Comparison of phylogenetic reconstruction of the sampled tree species using the *matK* chloroplast gene (**A**) and the sample groupings from the PLS analyses at the host tree family level (**B**) and the host tree species level (**C**). Host tree families have been colored for clarity, and the sample countries have been barcoded to identify additional clustering trends. Branch numbers on A represent ultrafast bootstrapping results (1000 replicates).

To determine how the clustering of the PLS analyses compared to the phylogeny of the tree species, a phylogenetic tree of all sampled tree species was constructed using the *matK* chloroplast gene (Figure 7, Table 2). This phylogeny was compared to the sample clustering observed in Figure 5 (host tree species) and Figure 6 (host tree family). Interestingly, the phylogenetic tree was nearly identical to the clustering of samples obtained from Figure 6 (host tree family), suggesting that the variation in the selected features was largely caused by the host tree. In contrast, the clustering of samples obtained from Figure 5 (host tree species) is inconsistent with the phylogenetic tree, suggesting that other factors may be influencing the variation of the selected features. To explore this, the sample clusterings presented in Figure 7 were labeled with the sample country. This revealed that some of the sample variations in Figure 5 could be explained by the sample country (and the respective microclimates of each country), as some of the samples clustered based on country of origin as opposed to taxonomy. As such, the features selected in Figure 6 were considered potential phytochemical responses to the host tree itself.

## 3. Discussion

In this work, it was observed that both geographic location and the type of host tree have the largest impact on the metabolites produced by *Radula complanata*. The different factors that may have contributed to these metabolomic shifts are discussed below.

At the geographic level, we found that the majority of the detected metabolites involved primary metabolites, specifically amino acids or peptides. This finding is in line with previous studies that found geography and climatic conditions to significantly shape plant populations by genetic variations [25,26]. These genetic variations can cause changes in gene expression, protein synthesis, and, ultimately, the production of metabolic compounds. Changes in gene expression [27] and protein production [28] occur in response to environmental stresses and are part of the way isolated populations adapt to their unique environmental conditions. Interestingly, the large difference in precipitation may account for the grouping observed in Figure 4, where Canada and Germany formed a clade while Sweden formed a separate group. Changes in protein expression have been observed in *Phoenix dactylifera* L., which has been experiencing drought [28], and amino acids were increased in drought-stressed tall fescue [29]. Similarly, *R. complanata* may experience a shift in protein and amino acid production when precipitation is limiting. Since *R. complanata* prefers wet environments [23], a low amount of precipitation (approx. 50 mm per month) could induce a significant drought response that is observable in the metabolome. It was also noted that the sampling sites in Sweden had higher wind speeds than the sites in Germany and Canada, which could have also resulted in the metabolite fluctuations observed. Increased wind speed may be associated with lower humidity levels [30], which could, in turn, contribute to a drier microclimate and the drought response of *R. complanata*.

Another factor that could be contributing to the metabolite variation is the proximity of each sampling site to a roadway. Roadways produce a variety of different air pollutants, including NO_X_, particulate matter, SO_2_, and ozone [31]. Epiphytic bryophytes are very sensitive to airborne pollutants and may be impacted by the pollutants emitted around roadways [22]. Unfortunately, each tree species occurs at a different distance to the nearest roadway, so it is unclear whether the observed changes are due to the host tree or to the air quality. Future work could be done to examine the impact of airborne pollutants on the metabolism of epiphytic liverworts.

Our study revealed a large impact of the host tree on the metabolic constitution of *R. complanata*. Although some of the explained variations are being shared and confounded by geographic location and likely related to different micro-climatic conditions (see above), the larger amount was attributed to the host tree species. This is likely due to the effect that many trees possess specific bark chemistry and often also have unique water retention capabilities that keep micro-climatic conditions rather stable [32,33]. Thus, environmental fluctuations are buffered, which gives epiphytic species a more stable environment to grow.

We observed a significant phylogenetic relationship of host tree species with the metabolic constitution of *R. complanata* independent of geographic origin. It has been described before that different host trees possess specific bark chemistry with unique conditions, such as bark pH or the elemental (C, N, P, Mg, Ca, …) content per bark dry mass filter for specific epiphytic communities [34,35]. While *R. complanata* has been found to be not very selective regarding its substrate (*R. complanata* can grow on many types of bark, mainly of deciduous trees, but also on rocks and even on bare soil), these unspecific and rather wide requirements are mirrored by its metabolome, which is regulated depending on the conditions of the host substrate. We found the majority of compounds associated with the host tree, either at the species level or the family level, to be specialized metabolites, such as glycosides, flavonoids, or benzenoids. However, we cannot completely rule out compounds that may be contributed by endophytes or other endogenic fungi to a small degree. As *R. complanata* is growing together with other epiphytic species in a community, such as epiphytic lichens, fungi, or other microbials, it is also likely that some metabolites are exchanged either between the other species in the community or the host tree itself. *R. complanata* may also produce metabolites specifically for the purpose of interacting with other species or the host tree. Assessing the number and diversity of these compounds may shed some more light on the status of epiphyte communities and their role in ecosystems. In general, it is hypothesized that the higher the diversity of compounds attributed to species interactions, the more stable the ecosystem and, hence, also the nutrient turnover and primary production [36]. Although our experimental design did not include the assessment of other species, we suggest that future studies could specifically target this central biodiversity hypothesis. Additionally, analysis of trees within the same family could also allow for more specific comparisons by limiting the variables contributing to the shifts in metabolites.

## 4. Conclusions

*Radula complanata* is a widely distributed species in the Northern hemisphere. It generally prefers places with medium to high air humidity (i.e., in the vicinity of waters), whereas the bark of trees is most often covered. When compared to other epiphytic bryophytes (such as *Frullania* spp., *Metzgeria* spp., *Orthotrichum* spp., …), it has a rather large amplitude and less specific requirements. This fact is mirrored by a highly flexible metabolome which we found to be adapted in response to the different conditions in the micro-climate on the bark of the host tree. We attributed the metabolomic shifts mainly to specialized metabolites, such as flavonoids, benzenoids, or several glycosylated compounds, which can serve as biomarkers regarding the individual fitness of *R. complanata* and may be mirroring the conditions in the surrounding habitat. We conclude that future studies should investigate whether rare or specialist species with narrower ecological requirements also have a less flexible and yet more specialized metabolome when compared to more generalist species such as *R. complanata*—and whether this observation can also be found in other groups, such as vascular plants. We found that the metabolic status of the epiphytic liverwort *R. complanata* can also mirror the constitution of the habitat, including the host tree, species interactions, and environmental conditions and that this information is very useful to develop conservation measures. However, dedicated studies are needed to further investigate the metabolic potential of epiphytic bryophytes. Further studies standardizing the conditions (such as trunk size, bark texture, sample height, and sample exposure) could also provide more insight into the specific environmental factors influencing the observed metabolic shifts.

## 5. Materials and Methods

### 5.1. Plant Collection

Plant material was harvested in the summer of 2021 from Sweden, Germany, and Canada (Table S4). Mature plants were collected and transported back to the lab, where the gametophytes were separated and frozen in liquid nitrogen. Plants were stored at −20 °C until HPLC analysis. Voucher specimens were deposited under the following accession numbers: JE04010995 to JE04011000 and UNB-UH-69080 to UNB-UH-69083.

### 5.2. HPLC Analysis and Raw Data Collection

The procedure from Blatt-Janmaat et al. was followed with no modifications [37]. Briefly, frozen plants were extracted with 80:20 MeOH:H2O, and samples were reconstituted to 10 mg fresh weight/100 µL for LC-MS analysis. Samples were separated using a Bruker Elite HPLC coupled to a Bruker TIMS-TOF equipped with a C18 column. A gradient of 0.1% aqueous formic acid and 0.1% acetonitrile was used as the mobile phase, and data was collected in the data-dependent acquisition (DDA-MS) mode. Raw LC/MS files (Bruker Daltonics .d format) were converted to .mzML with MSConvertGUI version 3 from the ProteoWizard software suite [38]. Raw data has been deposited to MetaboLights as MTBLS6740 (https://www.ebi.ac.uk/metabolights/MTBLS6740 (accessed on 22 January 2023)) [39].

### 5.3. Peak Detection and Data Treatment

The procedure for peak detection was obtained from Blatt-Janmaat et al. [37]. Any modified parameters will be listed; however, no major modifications were made. Briefly, R 4.1.1 (available from https://cran.r-project.org/ (accessed on 22 January 2023)) with the XCMS 3.14.1 package [40] was used for peak detection. Parameters were optimized with IPO [41], and manual adjustments were made based on instrument knowledge. The centWave algorithm was applied for peak detection [42] with the following adjusted parameters: ppm = 9.5, mzCenterFun = “wMean”, peakwidth = c(4, 33), prefilter = c(2, 140), mzdiff = 0.0012, noise = 0, integrate = 1, firstBaselineCheck = TRUE, verboseColumns = FALSE, fitgauss = FALSE, roiList = list(), roiScales = numeric(). Additionally, snthresh = 10 for negative mode and snthresh = 5 for positive mode. Detected peaks were grouped (parameters: minFraction = 0.7, bw = 2), and retention time was corrected using the adjustRtime (parameters: minFraction = 0.7, smooth = “loess”, span = 0.2, family = “gaussian”) function in XCMS, and finally, peaks were filled with the fillChromPeaks function in XCMS. Data were quality checked manually by extracting internal standard peaks and analyzing the shape and retention time of the peaks. Positive and negative data sets were independently processed and merged into a combined feature table which was used to construct several other constrained tables. A presence/absence table was constructed to determine if a peak was present in the MS1 data (cut-off 0.1% of maximum peak), and a compound table containing features that had MS2 spectra available was also constructed. Data tables are available in MetaboLights as MTBLS3563. In preparation for statistical analysis, missing MS1 data were replaced with the value 0.1. MS2 spectra were extracted from MS1 spectra and imported to SIRIUS 5.5.7 for further analysis.

### 5.4. Statistical Analyses

All statistical testing was conducted in R. Variation partitioning was conducted with the varpart function from the vegan package to identify key factors influencing the data. Sample separation was visualized by conducting Principal Component Analyses (PCA) using the prcomp function. PLS analysis was conducted to select those variables that are most different between the samples [43].

### 5.5. Compound Annotation and Classification

Peaks were tentatively classified and annotated using the SIRIUS software version 5.5.7, which contained SIRIUS [44], ZODIAC [45], CSI:FingerID [46,47], and CANOPUS [48,49]. Default settings were used for SIRIUS, ZODIAC, and CSI:FingerID, however only formulas from natural-product-based databases were considered (Bio Database, Biocyc, CHEBI, COCONUT, EcoCyc Mine, CNPS, HMDB, KEGG, KEGG Mine, KNApSAcK, Natural Products, and Plantcyc) for CANOPUS. Manual analysis of the SIRIUS, ZODIAC, CSI:FingerID, and CANPOUS scores was conducted to assign the selected features.

### 5.6. Phylogenetic Analysis

To identify the taxonomic groupings of the host tree species, a phylogenetic tree was constructed using the chloroplast maturase K (*matK*) gene. DNA sequences were collected from NCBI and aligned with MAFFT version 7 [50,51,52,53]. The IQ-TREE [54] webserver was used for model selection, tree construction, and branch support. Aligned sequences were tested for a substitution model with ModelFinder [55], a maximum likelihood tree was constructed [56], and ultrafast bootstrapping [57] was conducted to provide branch support. Default parameters were utilized for everything in IQ-TREE.

### 5.7. Climate Data

The climate data was downloaded from WorldClim2 [24] and extracted with the raster [58] package in R. Data from May to September of 2010–2018 were averaged for each region to generate average summer values for each weather variable (min temperature, max temperature, and precipitation) (Table S5). This data was plotted to generate climate maps and outlined using the maps package in R. Geographic coordinates from each sampling site were used to extract site-specific average values from the recent (2010–2018) (Table S2) and historical weather data (1970–2000) (Table S6). The spatial resolution for the historical weather data was ~1 km^2^, while the spatial resolution for the 2010–2018 data was ~21 km^2^.

## Figures and Tables

**Table 1:** Average summer (May-September) weather data for the sampling sites from 2010–2018.

Sample	Min Temperature (°C)	Max Temperature (°C)	Precipitation (mm)
GER-1	7.693678	18.45839	115.9697
GER-2	7.693678	18.45839	115.9697
GER-3	7.693678	18.45839	115.9697
SWE-1	10.89388	19.29325	46.78941
SWE-2	10.5323	19.41664	45.9303
SWE-3	10.11017	19.82523	49.68862
CAN-May	10.66182	23.49243	97.7621
CAN-June	10.35292	23.09298	97.39983
CAN-Aug	10.35292	23.09298	97.39983

**Table 2:** Sequence data used for building the phylogenetic tree.

Accession Number	Species	Voucher
KX676526.1	*Betula pendula* Ehrh. Roth	HERB0072
KX230018.1	*Scandosorbus intermedia* (Ehrh.) Sennikov	OMHD19M
JQ412285.1	*Sambucus nigra* L.	BS0146
KX677621.1	*Quercus robur* L.	HERB0346
KX229854.1	*Alnus glutinosa* (L.) Gaertn.	OMHD05M
KX229847.1	*Acer pseudoplatanus* L.	OMHD21M
EU749296.1	*Betula papyrifera* Marshall	OAC:JAG175
HQ593376.1	*Ostrya virginiana* (Mill.) K.Koch	AP243

## Data Availability

Raw data has been deposited to MetaboLights as MTBLS6740 (https://www.ebi.ac.uk/metabolights/MTBLS6740. Source code to recreate the results has been made available on GitHub (https://github.com/ipb-halle/iESTIMATE/tree/main/use-cases/radula-environmental).

## Supplementary Materials

The following supporting information can be downloaded at: https://www.mdpi.com/article/10.3390/plants12030571/s1, Table S1: Sampling data, Table S2: Climate data for 2010-2018, Table S3: Climate data for 1970-2000, Table S4: Feature identification for Figure 4, Table S5: Feature identification for Figure 5, Table S6: Feature identification for Figure 6.
